# Accuracy of Photon Dose Calculation on Photon-Counting Computed Tomography—A Comparison Study Based on Virtual Monoenergetic and Electron Density (Rho) Images for Pancreatic Cases

**DOI:** 10.1016/j.adro.2025.101957

**Published:** 2025-11-19

**Authors:** Xin Wu, Patrick Wohlfahrt, Jainil Shah, Ludovica Lofino, Manisha Palta, Qiuwen Wu, Q. Jackie Wu, Yang Sheng

**Affiliations:** a Department of Radiation Oncology, Duke University Medical Center, Durham, North Carolina; b Siemens Healthineers, Varian, Cancer Therapy Imaging, Forchheim, Germany; c Siemens Healthineers, Varian, Cancer Therapy Imaging, Cary, North Carolina; d Department of Radiology, Duke University Medical Center, Durham, North Carolina

## Abstract

**Purpose:**

Photon-counting computed tomography (PCCT) offers versatile anatomic information because of better energy discrimination and higher spatial resolution than conventional energy-integrating computed tomography (CT). With its rapid applications in diagnostic imaging, its potential within radiation oncology remains largely unexplored. Successful radiation therapy (RT) relies on both high-quality images to delineate tumor volume and accurate physical information of the anatomy for dose calculation. Specific to RT, dose calculation stands as a pivotal component in the process of treatment planning. This proof-of-concept study aimed to assess the accuracy of dose calculation and build the benchmark using PCCT images from a clinically operating NAEOTOM Alpha PCCT scanner from Siemens Healthineers.

**Methods and Materials:**

A total of 29 patients receiving abdominal contrast-enhanced PCCT scans were included in this study with institutional review board approval. The following 2 sets of reconstructed images were selected for this study: (1) virtual monoenergetic images (VMIs) of 70 keV, which resembles the conventional CT image at 120 kVp, and (2) electron density (Rho) images derived from PCCT, which provides direct physical information for photon dose calculation. In addition to the default Hounsfield look-up table (HLUT) implemented in a treatment planning system, which is suitable for conventional CT images, a specific HLUT converting 70 keV VMI CT numbers to relative electron density was established following the consensus guidelines. Patient’s organs were contoured using a deep-learning-based autosegmentation model in syngo.via RT Image Suite from Siemens Healthineers. An elective and a gross tumor volume were simulated in the pancreatic region for treatment planning within this study. All patients were planned with a 9-field intensity modulated RT with simultaneous integral boost regimen 25/33 Gy using an in-house deep-learning-based autoplanning model. Planning guidelines followed the institutional pancreas stereotactic body RT protocol. Eleven dose-volume histogram (DVH) metrics were included for comparison of 3-dose calculation approaches (conventional, 70 keV VMI and Rho).

**Results:**

The results revealed minimal dosimetry differences between 70 keV VMI and Rho images, with DVH metric percentage differences predominantly within 1% range (max: -1.60%) and 3-dimensional gamma analysis (1 mm/1%) pass rate >98% for most cases. The patient with the largest differences showed an average DVH metric difference of -1.32% and 3-dimensional gamma pass rate of 94.82%. Overall isodose distribution remained similar between 2 images for each patient. Dose calculation using the default HLUT in Eclipse showed DVH differences within a 1.5% range.

**Conclusions:**

The results demonstrated good agreement in dose calculation between 70 keV VMI and Rho images. For regions with iodine contrast, Rho images provided by PCCT can suppress the contrast enhancement, thereby mitigating unnecessary uncertainties. This benchmark performance shows that PCCT can be safely used for dose calculation with certain advantages, making it a valuable alternative for RT treatment planning.

## Introduction

Photon-counting computed tomography (PCCT) has become a revolutionary advancement in x-ray medical imaging. The photon-counting detectors in principle can detect each photon separately with submillimeter resolution. They categorize each photon into an energy bin corresponding to its energy, creating a multienergy spectrum for each detector pixel.^1^ These data are used to generate high-resolution images that offer enhanced contrast and superior abilities in differentiating material composition compared with conventional energy-integrating computed tomography (CT) scanners. Because of its enhanced precision, improved signal-to-noise ratio^2^ and lower patient dose exposure,^3^ PCCT is quickly being translated from preclinical trials to routine clinical practices^4^ such as cardiovascular imaging,^5^ neurovascular imaging,^6^ angiography^7^ and lumbar spine imaging.^8^ Early studies have shown its potential advantages in accurately diagnosing various diseases and identifying tumors.^9^

With the rapid incorporation of PCCT into the realm of diagnostic imaging since its US Food and Drug Administration approval in September 2021,^10^ its natural expansion of application in radiation oncology remains a relatively underexplored area of research. PCCT’s enhanced image contrast capabilities, particularly in soft tissues,^5^ could potentially revolutionize radiation treatment and planning workflow in radiation oncology. Simard et al^11^ investigated PCCT’s potential improvement on generating virtual noncontrast images where photon dose could be calculated accurately. Their simulation conducted on a virtual patient revealed that using PCCT with 4 energy bins reduced the root-mean-square (RMS) errors of virtual noncontrast images compared to the results obtained using dual-energy CT (DECT). RMS for the electron density and stopping power decreased from 2.0% to 1.4% and 2.7% to 1.4%, respectively. Hu et al^12^ assessed the key parameters for proton therapy of a head phantom, including the relative electron density and effective atomic number which were used for calculating proton relative stopping power. Their findings in this phantom using an in-house tool revealed an RMS error of 1.52%, 2.90%, and 1.27% for relative electron density, effective atomic number, and relative stopping power, respectively, which were comparable with DECT. The 2 studies have demonstrated PCCT’s capability to yield accurate electron density information that is crucial for photon dose calculation. Nevertheless, to the best of our knowledge, there has been no prior research directly investigating the end-to-end accuracy of dose calculation using PCCT in the context of treatment planning.

DECT and PCCT can generate high-quality virtual monoenergetic images (VMI)^13^ as well as accurate electron density images (Rho),^14^ which conventional single-energy CT simulators are not capable of. Both VMI and Rho images are viable options for dose calculation in radiation treatment planning.^15^ However, the benchmark performance of accuracy on a commercial treatment planning system (TPS) has not been established. Therefore, this study aims to assess the accuracy of dose calculation using PCCT images from a NAEOTOM Alpha PCCT scanner (Siemens Healthineers, Forchheim, Germany). The Hounsfield look-up table (HLUT) specifically tailored for PCCT-derived 70 keV VMI was measured and used for dose calculation. The precision of this method was evaluated by comparing the calculated doses against the “ground truth” (GT) doses calculated directly from PCCT-derived Rho images. Furthermore, we explored a “rapid alternative” calculation approach that involves the application of a conventional CT simulator HLUT to PCCT images. The accuracy of this HLUT is verified on all CT scanners in our institution and is used as the default HLUT in the TPS. In this study, the PCCT images were used as planning images within the treatment planning workflow to demonstrate end-to-end performance benchmarks. To our knowledge, this proof-of-concept study is the first attempt to assess the accuracy of photon dose calculation on actual patient’s plan using PCCT images.

## Methods and Materials

### Patient cohort and treatment planning

In this study, a total of 29 patients receiving abdominal contrast-enhanced PCCT scans at Duke University were included with institutional review board approval. VMI of 70 keV and electron density images (Rho) were reconstructed from spectral postprocessing data by using linearly weighted combination of low- and high-energy CT information.^16^ The selection of 70 keV VMI in this study is consistent with prior pancreas imaging protocols using PCCT and is equivalent to 120 kVp conventional CT in terms of attenuation characteristics as supported by multiple studies.^17^, ^18^, ^19^ Throughout the remainder of this paper, when not explicitly stated otherwise, "PCCT" shall refer to the 70 keV VMI image set. PCCT can generate VMIs at various energy levels; however, the choice of 70 keV does not limit the scope of this study, as further elaborated in the Discussion section.

The HLUT converting PCCT CT numbers to relative electron density was established following the recently published consensus guidelines.^20^ This publication has provided a detailed step-by-step guide on obtaining an HLUT in clinic workflow, which includes scanning a calibration phantom with tissue-equivalent inserts, extracting CT numbers under clinical scan protocols, and correlating them with known relative electron densities to generate a piecewise linear HLUT. The HLUT for the Rho image is a direct conversion table. Both HLUTs were entered into the Varian Eclipse TPS (version 15.6, Varian Medical Systems).

For each patient, 2 simulated targets were delineated including a primary planning target volume (25 Gy) and a boost planning target volume (33 Gy) using pancreatic stereotactic body radiation therapy (SBRT) protocol. Patient’s organs were contoured using a deep-learning-based autosegmentation model using syngo.via RT Image Suite VB80 from Siemens Healthineers. All patients were planned with a 9-field intensity modulated radiation therapy with a simultaneous integral boost regimen using an in-house deep-learning-based autoplanning model^21^ to reduce planning bias. The artificial intelligence (AI) model was composed of 2 consecutive convolutional neural networks (CNNs), 1 field-dose CNN for field-dose estimation, and a fluence map CNN for final fluence prediction. The model training followed the established institutional planning guidelines for pancreas SBRT. The AI model feeds the input of organ projections for each beam and predicts 9 beams’ optimal fluence maps. The fluence maps were subsequently imported into the TPS to perform leaf sequencing and dose calculation. All beams used 10 MV photon beams. Eleven dose-volume histogram (DVH) metrics were included for comparison of 3 dose calculation approaches.

### Dose calculation

**Figure 1:**
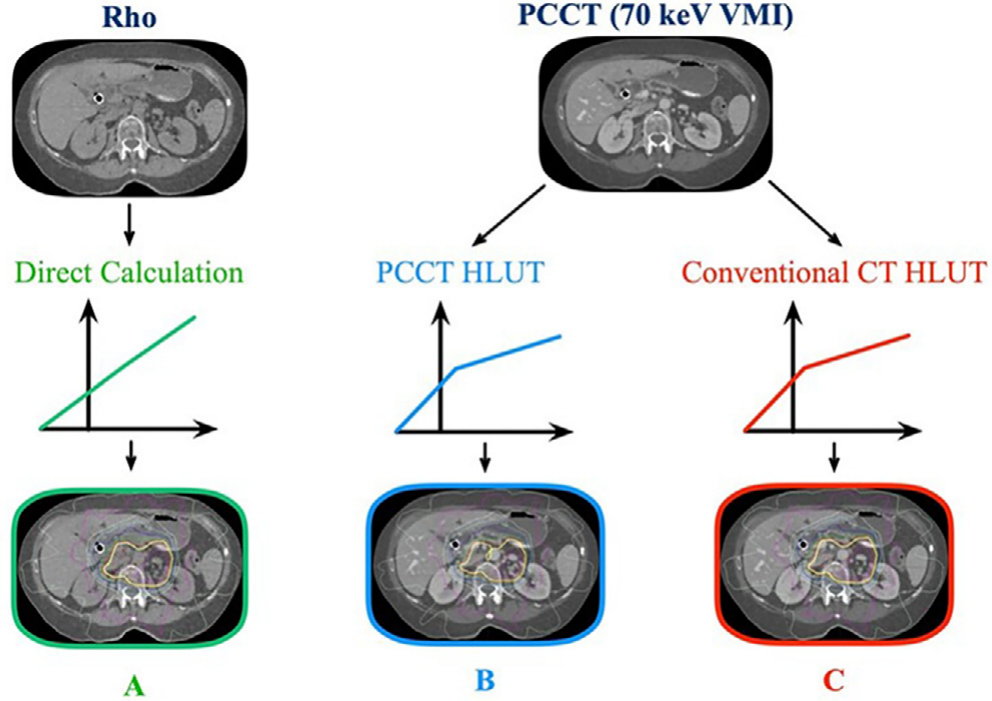
Three**-**dose calculation approaches. (A) Rho image with direct conversion. (B) PCCT with PCCT-dedicated HLUT. (C) PCCT with conventional CT default HLUT.

For each patient, we applied the following 3-dose calculation approaches shown in Fig. 1:

(1) Rho image with direct dose calculation (GT). Because Rho images derived from spectral information contain highly accurate electron density information,^14^ this set of dose distributions is referred to as GT.
(2) PCCT image with PCCT-dedicated HLUT.
(3) PCCT image with default HLUT for conventional CT (rapid alternative).

For each patient, the AI plan is calculated for all 3 approaches with the same optimal fluence maps and monitor units applied. Dose calculations were performed using the dose calculation algorithm in Varian Eclipse (Anisotropic Analytical Algorithm V15.6). Dose calculation scheme A used the electron density directly without any conversion, which was in principle the GT. However, in practical settings, physicists and physicians generally work with processed images instead of directly interacting with electron density images. Consequently, approach B, which uses PCCT images in conjunction with the corresponding empirically measured HLUT established for PCCT, is closer to the current clinical practice.

**Figure 2:**
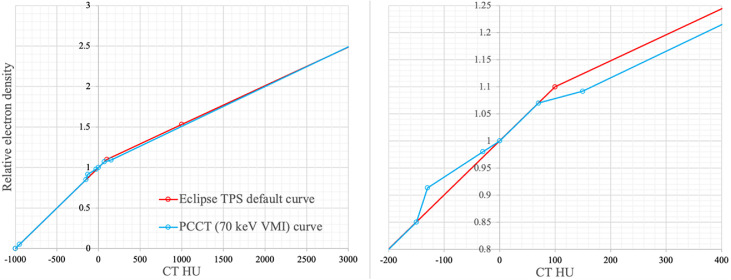
HLUT for PCCT and Eclipse TPS. Left: full CT number range; right: zoomed in sections (-200 to 400 HU) exhibiting the greatest degree of discrepancy.

Scheme C served as a “rapid alternative” version of dose calculation on PCCT, where the default HLUT established in the TPS was used. This institutional HLUT was commissioned based on measurements of a standard electron density phantom on multiple CT scanners with different scanning protocols and is in use for both research and clinical practice at our center. As shown in Fig. 2, the differences between 2 curves were generally very small. The region with the highest differences, between -200 HU to +400 HU as presented in Fig. 2, corresponds to the soft tissues. This observation justifies the inclusion of pancreatic patients in this study. Comparing the results of C with the GT will help assess whether the default HLUT in the TPS is suitable for dose calculation on PCCT in the absence of a tailored HLUT for that virtual energy.

### Accuracy assessment

For all 3 schemes, various dose metrics including $D_{\text{mean}},\;D_{98},\;D_{95},\;D_{0.1\text{cc}}$ were computed for 2 targets, whereas for the C-loop, the organ-at-risk (OAR), $D_{\text{mean}},\;D_{0.2\text{cc}},\;D_{0.1\text{cc}}$ were assessed. We defined the DVH metric relative difference (DMRD) as the discrepancy between the corresponding dose and the GT dose in scheme A:

$$\text{DMRD}=\frac{D_{x}-D_{A}}{D_{A}},\;x=B,\;C$$

DMRDs were calculated across all patients, yielding 11 DMRDs per patient and a total of 11 × 29 = 319 values for accuracy assessment.

For dose calculation results of schemes A and B, a 3-dimensional (3D) gamma analysis with a dose tolerance of 1% and a distance-to-agreement of 1 mm was implemented for all patients using the open-source software Plastimatch (V1.9.4). Original dose files with a resolution of $2.5\;\times \;2.5\;\times \;2\;mm^{3}$ were internally resampled to $1\;\times \;1\;\times \;1\;mm^{3}$. The analysis threshold was set to 10%, eliminating voxels with doses <10% in the 3D gamma analysis.

## Results

**Figure 3:**
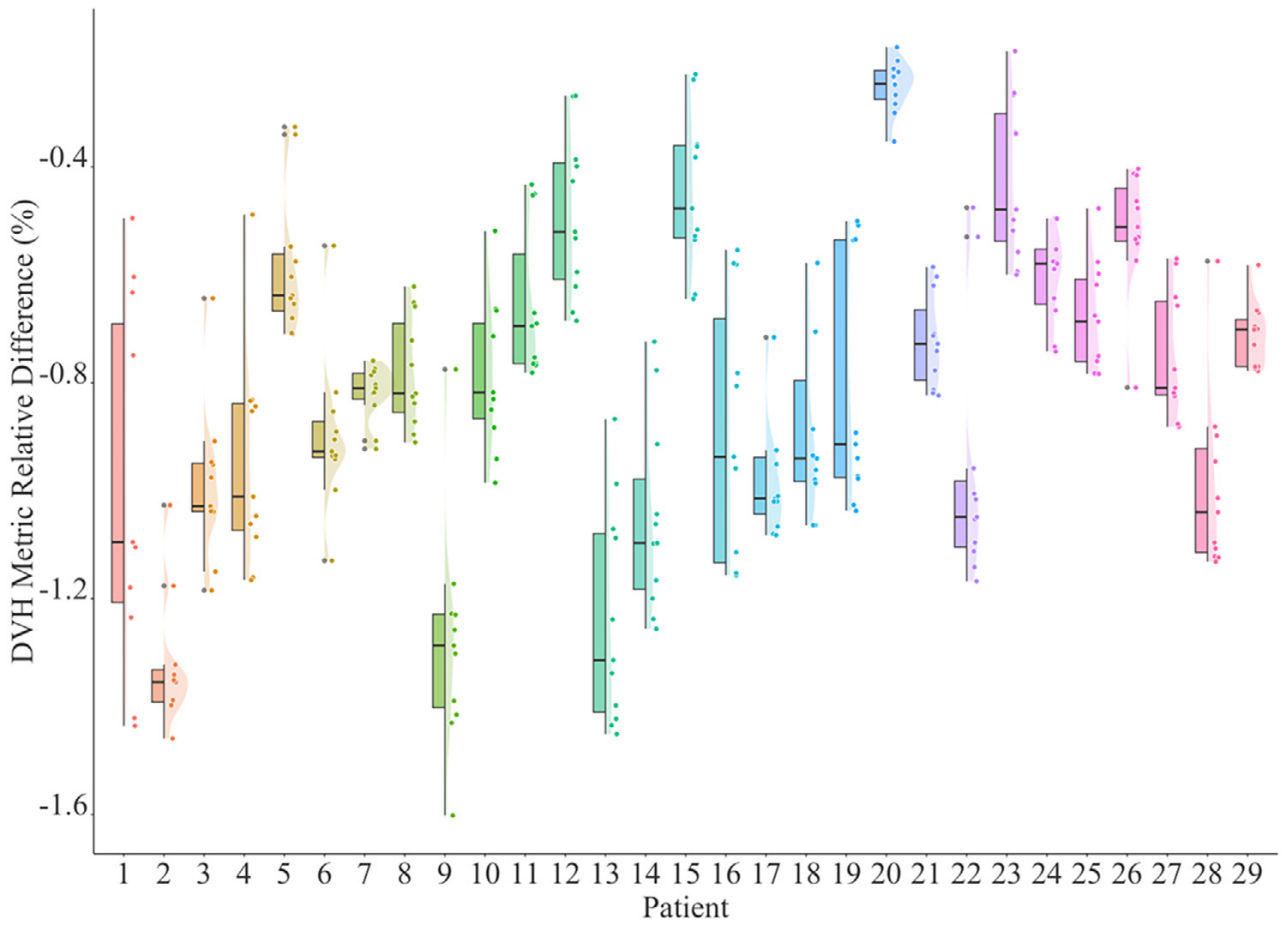
DMRD values for each patient calculated for dose differences of approach (A and B). Original data points were plotted with distributions (dots) and boxplots showing the quantiles.

First, results of approaches A and B were compared. For each patient, the DMRDs were calculated for all 11 DVH metrics. The values were plotted in Fig. 3 in 3 forms: original values (dot), distributions (curve shade), and boxplots. The mean values, SDs of DMRDs, and the 3D gamma analysis (1 mm/1%) pass rates for each patient were listed in column versus B’ of Table 1.

**Table 1:** Mean and SD for 11 DMRDs of each patient

	A vs B			A vs C		B vs C	
Patient	Mean (%)	SD (%)	3D gamma (%)	Mean (%)	SD (%)	Mean (%)	SD (%)
1	-0.97	0.34	97.90	0.08	0.48	-1.05	0.16
2	-1.32	0.12	94.82	-0.70	0.17	-0.63	0.12
3	-0.99	0.14	99.02	-0.38	0.16	-0.61	0.02
4	-0.94	0.20	98.61	-1.32	0.29	0.39	0.11
5	-0.58	0.13	100.00	-0.28	0.27	-0.30	0.14
6	-0.90	0.14	98.80	-1.09	0.23	0.20	0.09
7	-0.82	0.05	99.99	-1.15	0.09	0.33	0.05
8	-0.78	0.10	99.77	-1.18	0.19	0.41	0.11
9	-1.28	0.21	94.25	-1.74	0.27	0.46	0.07
10	-0.78	0.14	99.99	-0.76	0.28	-0.02	0.15
11	-0.66	0.14	100.00	-0.77	0.21	0.12	0.07
12	-0.49	0.15	100.00	-0.75	0.26	0.26	0.12
13	-1.24	0.20	94.93	-1.51	0.32	0.28	0.14
14	-1.05	0.18	96.73	-0.73	0.24	-0.33	0.08
15	-0.45	0.14	99.89	-0.16	0.18	-0.29	0.14
16	-0.89	0.24	99.49	-1.00	0.36	0.11	0.13
17	-0.98	0.10	98.61	-0.98	0.18	0.00	0.09
18	-0.89	0.16	99.82	-0.99	0.25	0.10	0.10
19	-0.80	0.23	99.31	-0.56	0.36	-0.25	0.15
20	-0.25	0.05	99.99	0.60	0.11	-0.85	0.08
21	-0.72	0.09	100.00	-1.09	0.19	0.37	0.11
22	-0.96	0.24	99.96	-0.41	0.28	-0.56	0.12
23	-0.42	0.15	100.00	-0.32	0.27	-0.11	0.15
24	-0.60	0.08	100.00	-0.30	0.13	-0.30	0.13
25	-0.68	0.10	100.00	-0.42	0.13	-0.26	0.10
26	-0.52	0.11	100.00	-0.09	0.20	-0.43	0.16
27	-0.75	0.12	100.00	-0.66	0.22	-0.09	0.12
28	-0.99	0.17	98.14	-0.90	0.29	-0.10	0.17
29	-0.71	0.06	99.97	0.10	0.08	-0.81	0.08
Mean	-0.81	0.15	98.97	-0.67	0.23	-0.14	0.11

The bottom row calculates the average of all 29 patients. For A vs B column, the 3D gamma analysis (1%/1 mm) pass rates are also presented.

**Figure 4:**
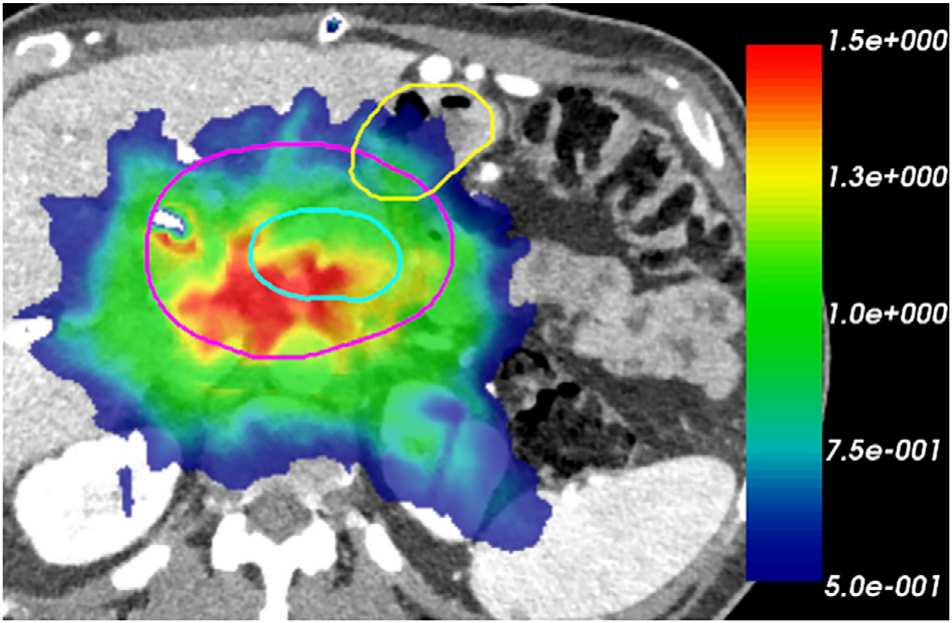
3D gamma index distribution for patient #2, same slice as Figure 4. Regions with iodine contrast enhancement are highlighted with white arrows. Contours: cyan: PTV33; purple: PTV25; yellow: C-Loop.

Figure 3 indicates that the individual DMRD predominantly fell <1% range (232 of 319 values, 73%). The maximum difference observed was -1.60% ($D_{0.1\text{cc}}$ of patient #9, Rho: 38.38 Gy vs PCCT: 37.76 Gy), which was also the only 1 in the 319 values that had a difference >1.5%. As shown in Table 1, 25 of 29 patients had mean DMRD under 1% and the highest mean DMRD was -1.32% (patient #2). For each patient, the 11 DMRD values also exhibited minimal variation with the highest SD of 0.34% (patient #1), which was an indication of a systematic deviation. The 3D gamma analysis pass rates were high despite the strict criterion (1mm/1%): with an average pass rate of 98.97% and 25 patients >98% and 14 patients even reached a 100% pass rate. The 3D gamma index distribution for patient #2, who exhibited the highest dose differences, is shown in Fig. 4. Almost all the high gamma index values were located in the target region.

**Figure 5:**
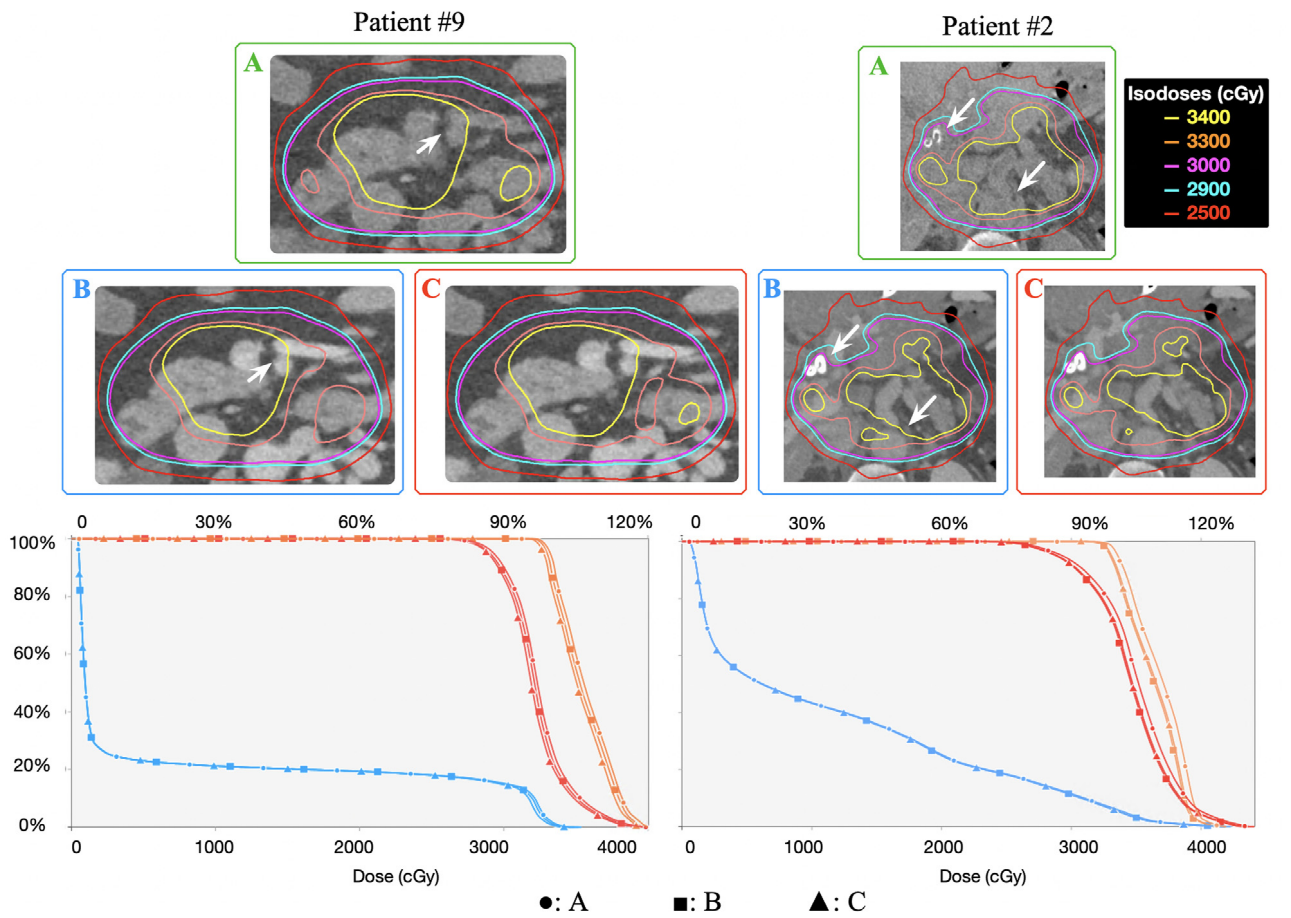
Dose distribution comparison for patients #9 and #2. First row: ground-truth dose using approach A; second row: dose distributions of approaches B and C; third row: DVH plot for PTV25 (red), PTV33 (orange), and OAR (blue) of 3 approaches. Regions with iodine contrast enhancement are highlighted with white arrows.

In Fig. 5, we highlighted 2 regions with the highest disparities in patient #9 and patient #2, which both showed the highest mean DMRD. As shown in the regions with white arrows, clear iodine contrast enhancement can be seen on the 70 keV VMI images (B, C) compared with Rho images (A), where it is significantly suppressed. In these regions, neighboring tissues exhibited relatively high isodose line discrepancies. The 3D gamma index (1 mm/1%) for patient #2 is shown in Fig. 4. For this patient, the aorta and stent regions, indicated by white arrows, showed contrast enhancement. The regions behind these areas exhibited large volumes of high gamma indices, comprising the majority of the unpassed regions.

When examining the detailed dose distributions, the isodose lines exhibited no discernible differences for the majority of cases. The overall isodose line shape remained largely preserved and was not clinically relevant. The only observable difference was a slight shrinkage of isodose lines of 34 Gy and 33 Gy observed in scheme B in Fig. 5, which was also reflected by the systematic shift of the DVH.

Differences in absolute dose values were also calculated for these simultaneous integral boost plans (25 Gy/33 Gy) for the 8 DVH metrics (total 29 × 8 = 232 values). The maximum absolute difference was 0.61 Gy and the minimum was 0.06 Gy. For each DVH metric, we also calculated the absolute dose difference averaged >29 patients. The mean difference value of the 8-dose metrics ranged from 0.26 Gy to 0.35 Gy.

### Accuracy of using default HLUT for PCCT dose calculation (“rapid alternative”)

**Figure 6:**
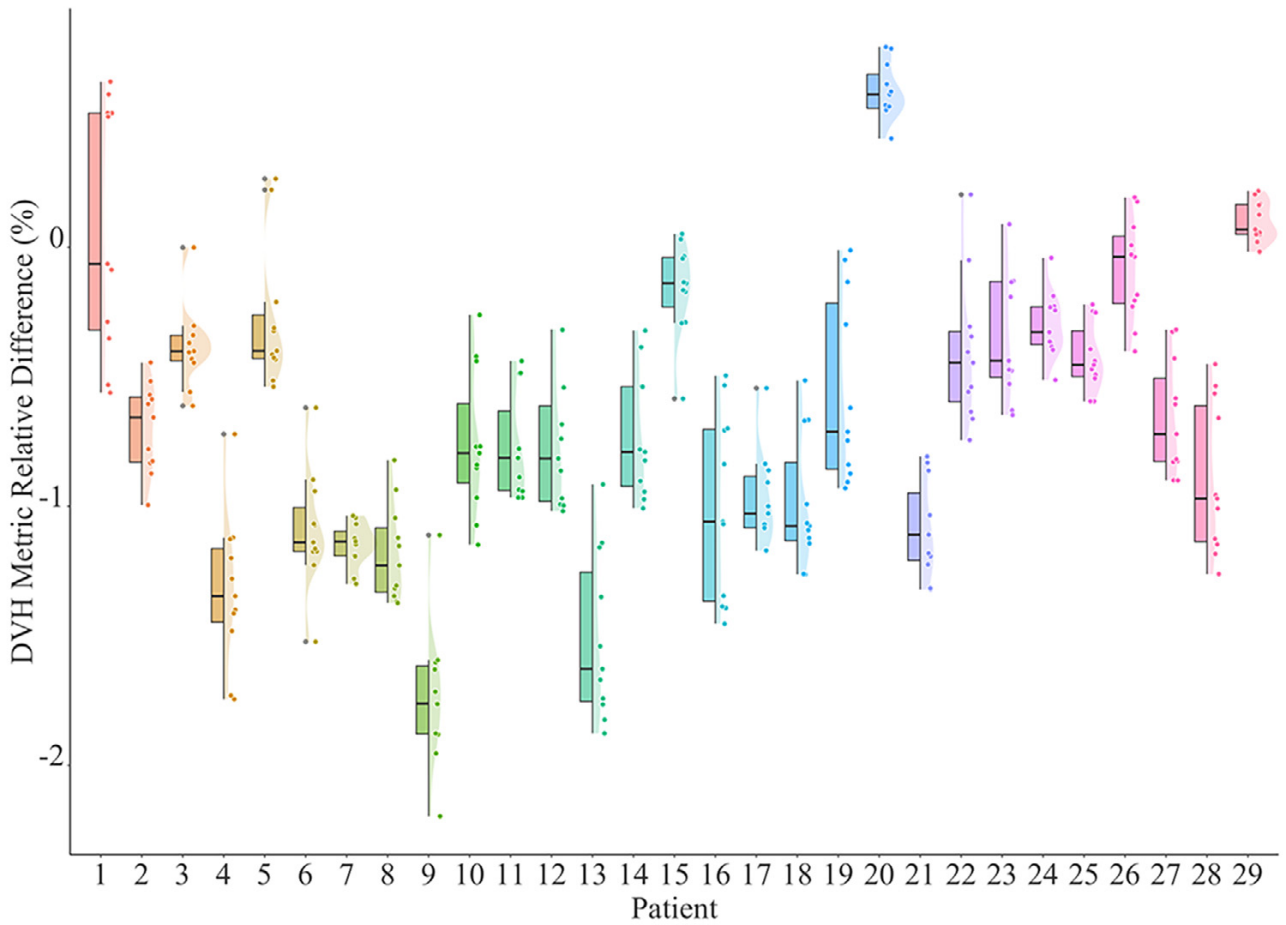
DMRD values for each patient calculated for dose differences between approaches (A and C). Original data points were plotted (dots) with distributions and boxplots showing the quantiles.

Second, dose results of A and C were compared. The DMRD was defined the same as the previous section and was shown in Fig. 6. The mean values and SDs for each patient were listed in column versus C’ of Table 1.

Compared with GT calculation in scheme A, the DMRDs for this rapid alternative scheme C had slightly higher variations. The highest value was -2.20% ($D_{0.1cc}$ of patient #9, A: 38.38 Gy, B: 37.54 Gy). A total of 223 of 319 values (69.9%) stayed under 1%, and there were 20 values that exceeded 1.5% compared with only 1 in scheme B. Patient-wise analysis showed that 22 of 29 patients had a mean DMRD less than 1% with the highest mean value of -1.74%. The isodose lines and DVHs were also presented in Fig. 5. Similar isodose line shrinkage and DVH shift were observed (particularly isodose line of 34 Gy).

## Discussion

This study investigated the dose calculation accuracy of using PCCT images for pancreas SBRT treatment planning workflow. The result of this study showed promising accuracy of using PCCT for photon dose calculation.

First, direct dose calculation on Rho images generated from spectral postprocessing data of PCCT was set as GT, which is supported by previous investigations on the accuracy of Rho images derived from spectral information provided by DECT and PCCT^.11,12,14^ Rho images can be safely used for dose calculation and treatment planning without commissioning an HLUT.

We emphasize that this study does not seek to demonstrate that PCCT offers superior dose calculation accuracy compared with traditional CT or DECT—although this may be true, it is not assessed in this work. The primary advantages of PCCT lie in its improved image quality, as outlined in the introduction, but its current use is limited to diagnostic imaging. Adopting PCCT for radiation therapy treatment planning requires dose calculation as the first step in the verification process. Therefore, the primary objective of this study is to establish the feasibility of performing dose calculations on PCCT with sufficient accuracy and to develop a benchmark workflow. To date, no prior studies have quantitatively investigated its dose calculation accuracy. This study aimed to fill that gap. With validated dose calculation accuracy, the advantages of PCCT—such as superior target delineation—can be fully leveraged for potential applications in radiation therapy.

Second, the comparison of DVH parameters and 3D gamma analysis between calculation results of Rho and HLUT approach does not present huge differences. Therefore, without direct operations on Rho images, approach B is close to the current practice for dose calculation on PCCT. Based on these results, it can be reported that the deviations from the GT dose are within 1.6% for key DVH metrics. High pass rates of the 3D gamma analysis with a strict criterion of 1mm/1% further demonstrate the minimal differences between the dose calculation results. Combined with its superior anatomic information, PCCT could be a versatile approach for the next generation of RT treatment workflow.

Third, Rho images can in principle suppress iodine enhancement, whereas conventional CT or VMI of 70 keV show iodine enhancement in organs, which translate into larger electron density values and thus introduce unnecessary dose calculation uncertainty in neighboring regions, especially the downstream volumes as shown in Fig. 5. Because Rho images can mitigate this, it is an advantage of incorporating PCCT in treatment planning. With PCCT, dose calculation can be internally implemented on Rho images and overlaid on high-resolution and high-contrast VMIs for physicists and physicians. This approach reduces the impact of contrast enhancement in dose calculation for improved accuracy while retaining the benefits of contrast in contouring and planning.

Another question was whether it is safe to apply approach C, the rapid alternative, when measured HLUT for 1 specific VMI energy is unavailable. Comparison between approaches B and C, as illustrated in the “B versus C” column in Table 1 and DVH depicted in Fig. 5, revealed slight discrepancies. This observation can be rationalized by recognizing that the sole distinctions between B and C lie in the HLUT as demonstrated in Fig. 2, which did not exhibit many differences. One can conclude that it is generally safe to apply approach C as an estimation with acceptable accuracy. However, it must be emphasized that even if the dose differences seem small, they still represent another unnecessary uncertainty that could be avoided. A dedicated HLUT should be measured, particularly when using specific energies of VMI in clinical scenarios.

In addition to its applications in photon plans, one potential advantage of PCCT for proton treatment planning is its ability to provide more accurate stopping-power ratio estimates, as indicated in previous studies.^12^ Furthermore, PCCT’s capability to remove contrast agents during image processing might be beneficial for protons as well, where dose calculations are highly sensitive to changes in material composition.

Although this pilot study demonstrates an end-to-end dose calculation workflow using PCCT-derived images, it also has some limitations, which we will further investigate in future follow-up studies. First, although this study focused on the 70 keV VMI, which is clinically relevant and commonly used to approximate conventional 120 kVp CT, PCCT is capable of generating VMIs across a wide range of energies—a key advantage over traditional CT. Future work will explore additional VMI energy levels to assess their impact on tumor and organ segmentation across different anatomic sites. However, different VMI energies will potentially offer great benefits in improved tumor and organ segmentation, where tissue contrast can be optimized and customized across different anatomic sites. For dose calculation, the electron density is the material quantity of interest for photon attenuation, and this is directly provided by the Rho images. Second, this study evaluated dose calculations within the abdominal region, which is an important clinical site that also demonstrates iodine contrast enhancement—an effect we proposed to mitigate using Rho images. However, challenges also persist in regions characterized by greater tissue heterogeneity, such as the lungs^22^ and head and neck cases.^23^ In these areas, VMIs of different energies are often used to optimize imaging quality.^13^ While the methodology we describe is fully generalizable to other anatomic regions, this work focused on a single treatment site, providing a clinically relevant but controlled first step before extending to other more heterogeneous anatomic regions. Future studies will expand on this work by investigating dose calculation accuracy in more anatomically complex regions, thereby advancing the understanding and clinical application of PCCT across a broader range of treatment sites. PCCT also currently comes with higher capital and operational costs. Similar to many new technologies being brought into radiation therapy workflow and practice, they were very expensive at the time of introduction. The strengths and benefits of photon-counting CT, include improved photon dose calculation accuracy, enhanced tumor and organ contouring, suppression of iodine contrast artifacts, and more importantly, the potential integration of functional information within a single acquisition; and its potential value for radiation oncology will need to be validated in prospective clinical studies. With the rapid evolution of the PCCT technology and more vendors bringing products to compete in the market, there are certainly projections that the cost of PCCT will drop in the future.

The result of this study demonstrated that PCCT offers comparable dose calculation accuracy compared to traditional CT. This is the first key step of proving the usability of PCCT in radiation therapy setting. In principle, the primary advantages of PCCT lie in its improved image quality, as outlined in the introduction, but its current use is limited to diagnostic imaging. We aim to seek the possibility of implementing PCCT in the radiation therapy workflow. Adopting PCCT for radiation therapy treatment planning requires dose calculation as the first step in the verification process. Therefore, the primary objective of the current study is to establish the feasibility of performing dose calculations on PCCT with sufficient accuracy and to develop a benchmark workflow. This work is the first attempt to investigate this topic. Subsequent studies will further investigate image quality and contouring capability in radiation therapy workflow.

## Conclusion

This work demonstrates the dose calculation accuracy of PCCT through the strong agreement observed between 70 keV VMI and Rho images. This benchmark performance confirms that PCCT can be reliably used for dose calculation, offering distinct advantages and positioning it as a valuable rival for radiation therapy treatment planning.

## Disclosures

Xin Wu M.S., Manisha Palta M.D., Qingrong-Jackie Wu Ph.D., and Yang Sheng Ph.D. received support from a Siemens Healthineers master research agreement.
